# All-trans retinoid acid promotes allogeneic corneal graft survival in mice by regulating Treg-Th17 balance in the presence of TGF-β

**DOI:** 10.1186/s12865-015-0082-3

**Published:** 2015-03-19

**Authors:** Xin Wang, Wentao Wang, Jianjiang Xu, Suqian Wu, Qihua Le

**Affiliations:** Department of Ophthalmology, Eye & ENT Hospital of Fudan University, Shanghai, 200031 China; Research Center, Eye & ENT Hospital of Fudan University, Shanghai, 200031 China

**Keywords:** All*-trans* retinoid acid, TGF-β, Th17 cells, Regulatory T cells, Corneal transplantation

## Abstract

**Background:**

All*-trans* retinoid acid (ATRA) has been proven to skew Regulatory T cell-T helper 17 cell (Treg-Th17) balance toward Treg *in vitro,* favoring graft acceptance. However, its *in vivo* effect after solid organ transplantation is under investigation.

**Results:**

BALB/c mice were given orthotopic corneal grafts from C57BL/6 donors, and recipient mice were administered with ATRA, TGF-β, and the combination of both agents for 8 weeks after surgery. We found that a mixed treatment of ATRA and TGF-β significantly promoted graft survival. Moreover, with the presence of TGF-β, ATRA upregulated CD4^+^CD25^+^Foxp3^+^Treg cells and suppressed Th17 cells in the blood, spleen and draining lymph nodes of recipient mice, as well as enhanced the Foxp3 expression and inhibited the RORγt expression in grafts and peripheral blood mononuclear cells (PBMCs). Simultaneously, increased number of Foxp3+ cells and decreased number of IL-17+ cells in conjunctiva were found in recipients with mixed treatment, along with reduced IL-17 level in serum and aqueous humor and increased IL-10 level in aqueous humor. Tregs isolated from recipient mice treated with ATRA + TGF-β presented the strongest suppressive activity *in vitro*.

**Conclusions:**

Combined application of ATRA and TGF-β may shift the Th17-Treg balance toward Tregs, hence facilitating the induction of immunological tolerance after allogenic corneal transplantation and representing a potential therapeutic approach in the treatment of posttransplant rejection.

**Electronic supplementary material:**

The online version of this article (doi:10.1186/s12865-015-0082-3) contains supplementary material, which is available to authorized users.

## Background

Corneal disease, one of the leading causes of blindness in China [1], requires corneal transplantation to restore visual function in the majority of cases [2]. Although immune privilege of corneal allografts endowed a higher success rate of corneal transplantation than other solid organ transplantation, immunological rejection is still the major cause of graft failure after penetrating keratoplasty [3,4].

CD4^+^CD25^+^Foxp3^+^Regulatory T cells (Treg), an important regulator in maintaining immune homeostasis, play a crucial role in protecting individuals from graft rejection [5]. In light of this, these cells are now considered an important target for generating tolerance to solid organ transplants. However, it has been found that Tregs can be subverted by inflammatory conditions and converted into interleukin-17 (IL-17)-producing T helper cells (Th17) [6]. Treg and Th17 cells share a common requirement for TGF-β in their differentiation, despite expressing distinct transcriptional regulators (Foxp3 versus RORγt, respectively) and demonstrating opposing functions. IL-17 is involved in the allograft rejection, and IL-17 antagonism prolongs graft survival [7,8]. The balance of Treg-Th17 axis and its shift toward the stabilization of Treg populations are one of the key points in maintaining graft tolerance.

Some cytokines or interventions have been proven to alter the Treg-Th17 balance. Either IL-6 or IL-27 could inhibit Treg induction and promote IL-17 production, shifting the balance of the Treg-Th17 axis toward Th17 [9,10]. However, the blockage of interferon regulatory factor 4 is beneficial in maintaining the Foxp3 expression, which is crucial for the immunoregulatory function of Tregs, and suppressing the production of IL-17 and IL-21, as well as prohibiting the activation of retinoic acid receptor-related orphan receptor (ROR) γt, the hallmark of Th17 activation [11].

Recent studies show that the key metabolite of vitamin A, all*-trans* retinoid acid (ATRA), potentiates TGF-β-dependent Treg induction, while it reciprocally inhibits pro-inflammatory Th17 differentiation *ex vivo* [12,13]. Disrupted ATRA metabolism or signaling causes altered homing or impaired functional differentiation of the lymphocytes [14]. ATRA has been demonstrated to sustain the stability of Foxp3 expression and suppress RORγt expression *in vitro,* maintaining the function of Tregs and inhibiting Th17 development [15,16]. However, the effect of ATRA on another pivotal transcriptional factor for the generation of Th17 cells, RORα, remains unclear.

Until now, regulation of the reciprocal differentiation of Tregs and Th17 cells has been investigated mainly *in vitro*. There have been few studies conducted on *in vivo* regulation. Moreover, few reports have been published to explore the effect of ATRA on Treg-Th17 balance after allogenic organ or tissue transplantation. Hence, we conducted this study to explore the effect of ATRA on Treg-Th17 balance after allogenic corneal transplantation and determine its correlation with the outcome of cornea grafts in recipient mice.

## Results

### A mixed treatment of ATRA and TGF-β promotes graft survival

**Figure 1:**
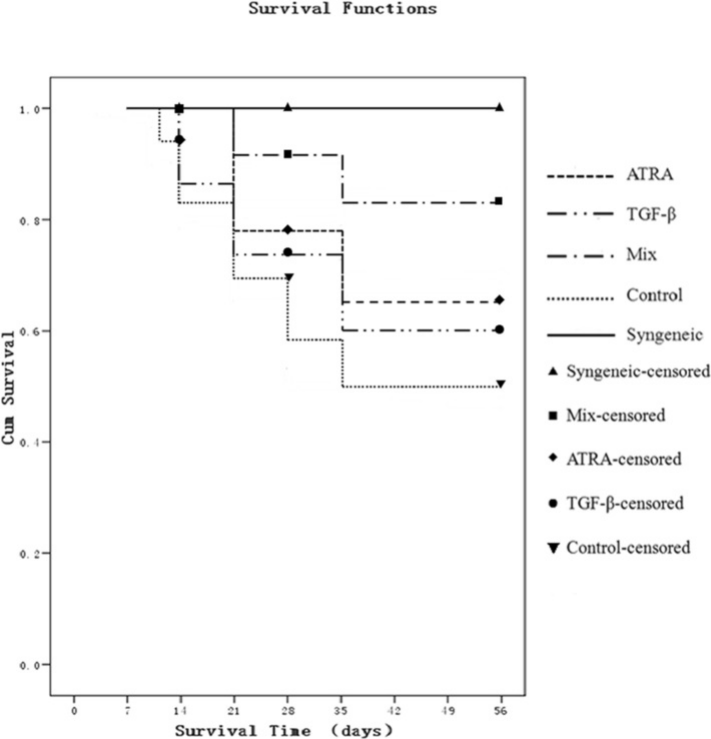
**Graft survival curve.** Each group contained at least 30 recipient mice at 14 days, 20 mice at 28 days and 10 mice at 56 days postoperative. The curve represents the average survival rate of each group. At the early time point (10–21 days), the grafts treated with either ATRA alone or the combination of ATRA and TGF-β had a modestly higher survival rate (100%) than those treated with a control solution (82%). Nevertheless, a single administration of TGF-β did not promote graft survival at an early stage following surgery. In contrast, at a late time point (35–56 days), the survival rate of grafts treated with either ATRA or TGF-β solely was significantly higher than controls (ATRA: 65% and TGF-β: 60% vs control: 48%). A mixed administration of ATRA and TGF-β further promoted graft survival rate to 82%.

During the follow-up, 9 mice died unexpectedly, two in ATRA group (at 8d and 35d respectively), one in TGF-β group (at 18d), three in ATRA + TGF-β group (at 11d, 24d and 42d respectively), two in control group (at 8d and 28d respectively) and one in synergistic group (at 39d). The same number of recipient mice was recruited. So a total of 159 mice were censored, with an overall graft survival rate of 71%. The recipient mice treated with the combination of ATRA and TGF-β presented a graft acceptance rate of 82% at the end of the 8-week follow-up, which is significantly higher than those treated with ATRA alone (P = 0.042), TGF-β alone (P = 0.023) and a control solution (P = 0.008). Meanwhile, it didn’t have significant differences compared with syngeneic grafts (P = 0.074), as shown in Figure 1.

### A mixed treatment of ATRA and TGF-β upregulates CD4^+^CD25^+^Foxp3^+^Treg cells in recipient mice

**Figure 2:**
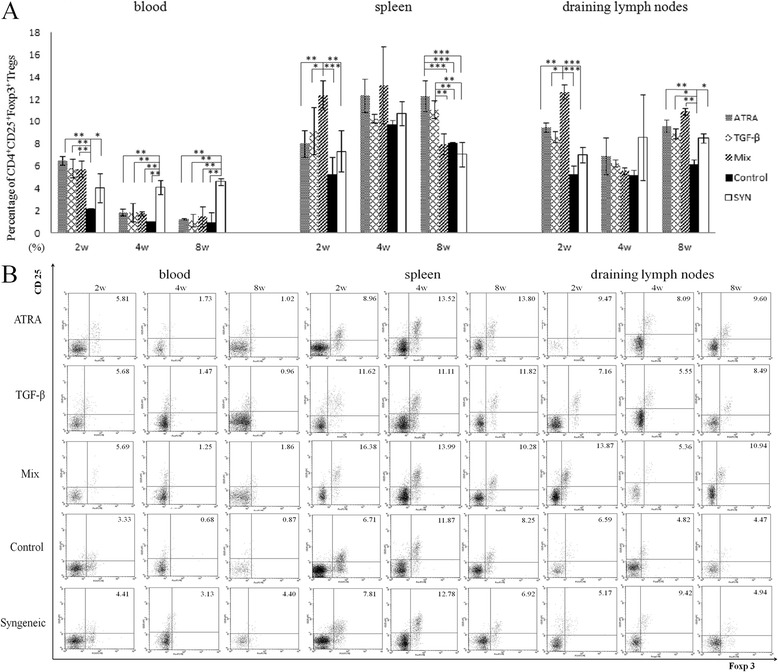
**Percentage of CD4** ^**+**^ **CD25** ^**+**^ **Foxp3** ^**+**^ **Treg cells in blood, spleen and draining lymph nodes. (A)** At 2wks postoperative, a single administration of either ATRA or TGF-β promoted the level of CD4^+^CD25^+^Foxp3^+^Treg cells in peripheral blood, spleen and draining lymph nodes, compared with those treated with a control solution. A mixed administration of ATRA and TGF-β further enhanced the level of Tregs. At 8wks postoperative, the level of CD4^+^CD25^+^Foxp3^+^ Treg cells in spleen and lymph nodes was increased in those with a single administration of either ATRA or TGF-β. Nevertheless, the synergistic effect was not found in those with a mixed treatment. Data are presented as the mean value ± standard error and are representative of at least three independent experiments. **(B)** Representative flow cytometric figures of Foxp3 and CD25 expression on CD3 + CD4 + cells from the blood, spleen and LN in recipient mice. * P < 0.05, ** P < 0.01, ***P < 0.001.

The percentage of CD4^+^CD25^+^Foxp3^+^Treg cells in blood was significantly increased at 2wks in recipients treated with ATRA, TGF-β and the combination of both agents (P = 0.020, 0.039 and 0.040, respectively). However, such effects were not found at 4wks and 8wks. Single treatment of either ATRA or TGF-β promoted Treg level in spleen at 2wks and 8wks (ATRA: P = 0.004 and 0.002, TGF-β: P = 0.001 and 0.025, respectively). Compared to recipients with a single administration, mixed treatment further enhanced Treg in the spleen at 2wks (vs ATRA: P = 0.023, vs TGF-β: P = 0.041). Nevertheless, the synergistic effect was not found at 4wks and 8wks. The results of Treg level in the lymph nodes were similar to those in the spleen, as shown in Figure 2.

### A mixed treatment of ATRA and TGF-β suppresses Th17 cells in recipient mice

**Figure 3:**
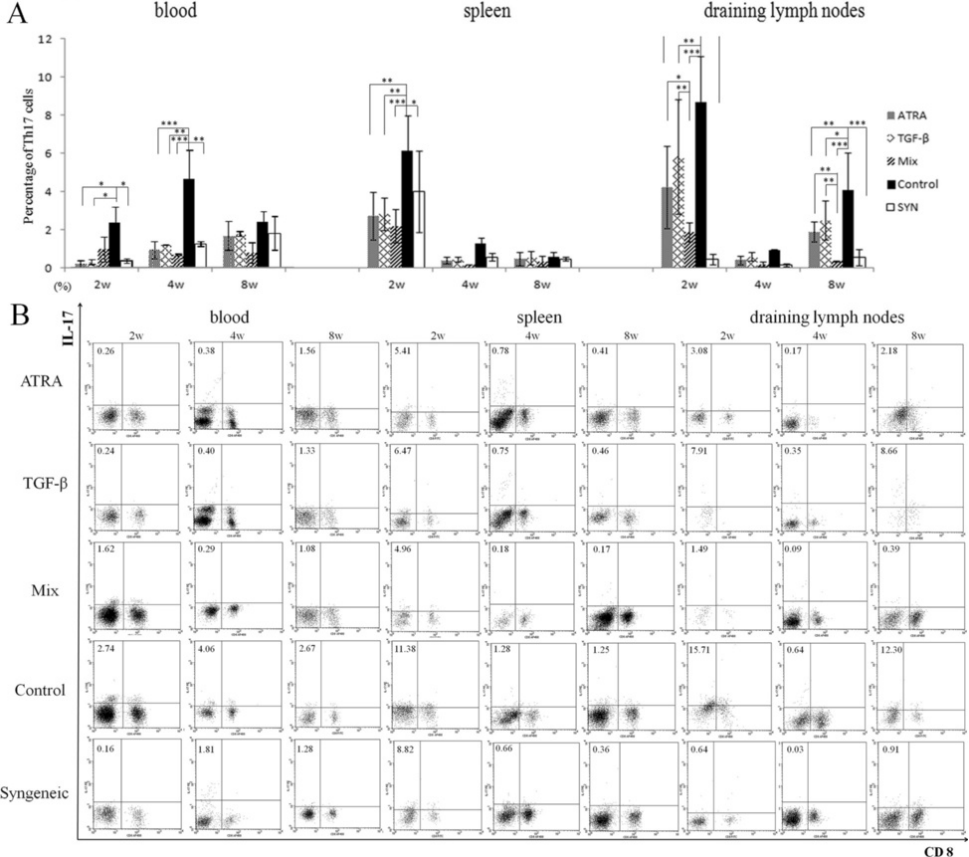
**Percentage of Th17 cells in blood, spleen and draining lymph nodes. (A)** At 2wks postoperative, the level of Th17 cells in recipients with a single administration of either ATRA or TGF-β was significantly lower than those treated with a control solution. A mixed treatment of these two agents could further suppress Th17 cells in lymph nodes at 2wks and 8 wks. Data are presented as the mean value ± standard error and are representative of at least three independent experiments. **(B)** Representative flow cytometric figures of IL-17 and CD8 expression on CD3 + CD4+ cells from the blood, spleen and LN in recipient mice. * P < 0.05, ** P < 0.01, ***P < 0.001.

The percentage of Th17 cells in blood was reduced significantly in recipients with a single administration of either ATRA or TGF-β at 2wks (ATRA: P = 0.011, TGF-β: P = 0.016). At 4wks, recipients with a mixed treatment also presented a significantly lower percentage of Th17 cells in the blood (ATRA: P <0.001, TGF-β: P = 0.001, mixed: P <0.001). The level of Th17 cells in the spleen was significantly higher in the control group than in the other four groups at 2wks (P <0.001). However, no significant differences were found among the five groups at 4wks and 8wks. A single treatment of either ATRA or TGF-β caused a significant reduction of Th17 cell levels in draining lymph nodes at 2wks and 8wks, compared with control recipients (both P <0.001). Moreover, a mixed treatment of ATRA and TGF-β further decreased the percentage of Th17 cells in the lymph nodes, as shown in Figure 3.

### A mixed treatment of ATRA and TGF-β increases Foxp3+ cells and decreases IL-17+ cells in conjunctiva, as well as prohibits inflammation in the grafts

**Figure 4:**
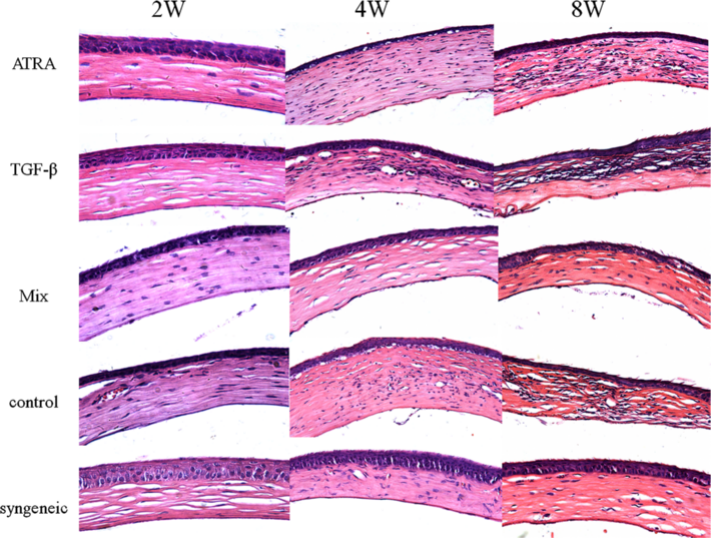
**Histology assessment of allogeneic grafts.** At 2wks after transplantation, no inflammatory infiltrates and graft edema were found in all groups. At 4wks, obvious inflammatory infiltration in the graft stroma and graft edema as well was visible in control solution-treated recipients. A smaller amount of inflammatory infiltration could be found in the TGF-β-treated recipients. However, neither grafts with mixed treatment of ATRA and TGF-β nor syngeneic grafts presented inflammatory infiltration and graft edema. At 8wks, more or less inflammatory cells were infiltrated in the graft treated with ATRA, TGF-β and control solution. In contrast, the lamellar structure of stromal collagen was clearly visible in grafts with a mixed treatment, despite epithelium proliferation. (Hematoxylin - eosin staining, × 200).

**Figure 5:**
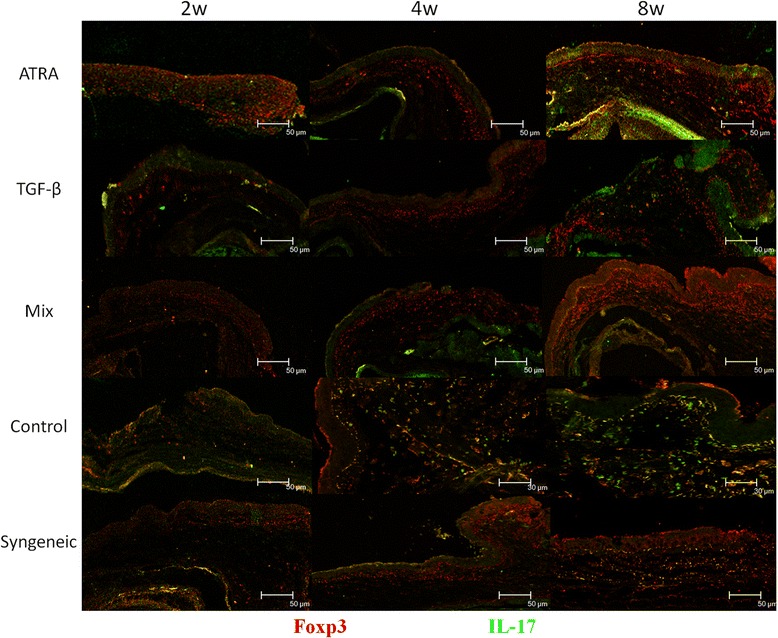
**Immunostaining of Foxp3+ cells and IL-17+ cells in recipients’ conjunctiva.** A few foxp3+ cells could be found in the conjunctiva of recipients treated with either ATRA or TGF-β at 2wks postsurgery. At 4wks and 8wks, Foxp3+ cells could be found in the conjunctiva of recipients treated with either ATRA or TGF-β, while IL-17+ cells were visible in control solution-treated ones. Recipients with a mixed treatment presented more Foxp3+ cells compared with those with a single treatment. Foxp3+ cells were stained as red and IL-17+ cells were green.

At 2wks after transplantation, no inflammatory infiltrates and graft edema were found in all groups (Figure 4, left column). For recipient mice treated with either ATRA or TGF-β, a few foxp3+ cells could be found in the conjunctiva (Figure 5, left column). At 4wks, obvious inflammatory infiltration in the graft stroma and graft edema was visible in the control solution-treated recipients. A smaller amount of inflammatory infiltration could be found in the TGF-β-treated recipients. However, neither grafts with a mixed treatment of ATRA and TGF-β nor syngeneic grafts presented inflammatory infiltration and graft edema (Figure 4, middle column). Foxp3+ cells could be found in the conjunctiva of recipients treated with ATRA, TGF-β or a mixture of both, while IL-17+ cells were visible in the conjunctiva of the control solution-treated recipients (Figure 5, middle column). At 8wks, more or less inflammatory cells were infiltrated in the graft treated with ATRA, TGF-β and control solution. In contrast, the lamellar structure of the stromal collagen was clearly visible in grafts with a mixed treatment, despite epithelium proliferation (Figure 4, right column). The immunofluorescence staining for conjunctiva presented similar results as compared with those taken at 4wks postoperative (Figure 5, right column). The quantitative analysis of inflammatory cells, Foxp3+ cells and IL-17+ cells in the tissue is presented in Table 1.

**Table 1:** **Quantitative analysis of inflammatory cells, Foxp3+ cells and IL-17+ cells in grafts and conjunctiva in recipient mice**

**(cells/μm** ^**2**^ **)**	**ATRA**	**TGF-β**	**ATRA + TGF-β**	**Control**	**Syngeneic**	**P value**
Inflammatory cells						
2w	0	0	0	14.9 ± 18.72	0	0.132
4w	496.1 ± 68.4	891.4 ± 80.9	93.2 ± 45.2	1842.5 ± 68.4	0	<0.001
8w	1356.0 ± 260.7	1843.2 ± 252.8	152.9 ± 52.9	1872.4 ± 106.3	29.8 ± 6.5	<0.001
Foxp3+ cells						
2w	21.9 ± 1.4	4.9 ± 0.6	16.7 ± 5.4	10.3 ± 2.7	25.6 ± 1.8	<0.001
4w	24.7 ± 0.6	29.4 ± 6.5	47.9 ± 2.2	22.2 ± 9.1	44.9 ± 6.7	0.004
8w	32.5 ± 5.1	36.9 ± 4.3	52.6 ± 4.8	25.5 ± 6.2	36.4 ± 7.3	0.001
IL-17+ cells						
2w	5.5 ± 1.0	8.5 ± 0.8	2.4 ± 0.4	8.1 ± 0.6	3.3 ± 1.2	0.017
4w	3.5 ± 2.3	3.5 ± 0.2	5.1 ± 1.6	46.7 ± 12.4	5.1 ± 0.8	<0.001
8w	7.9 ± 1.7	8.7 ± 1.2	2.9 ± 1.3	50.0 ± 4.8	3.8 ± 1.1	<0.001

### A mixed treatment of ATRA and TGF-β increases the Foxp3 expression and inhibits the RORγt expression in grafts and PBMCs

**Figure 6:**
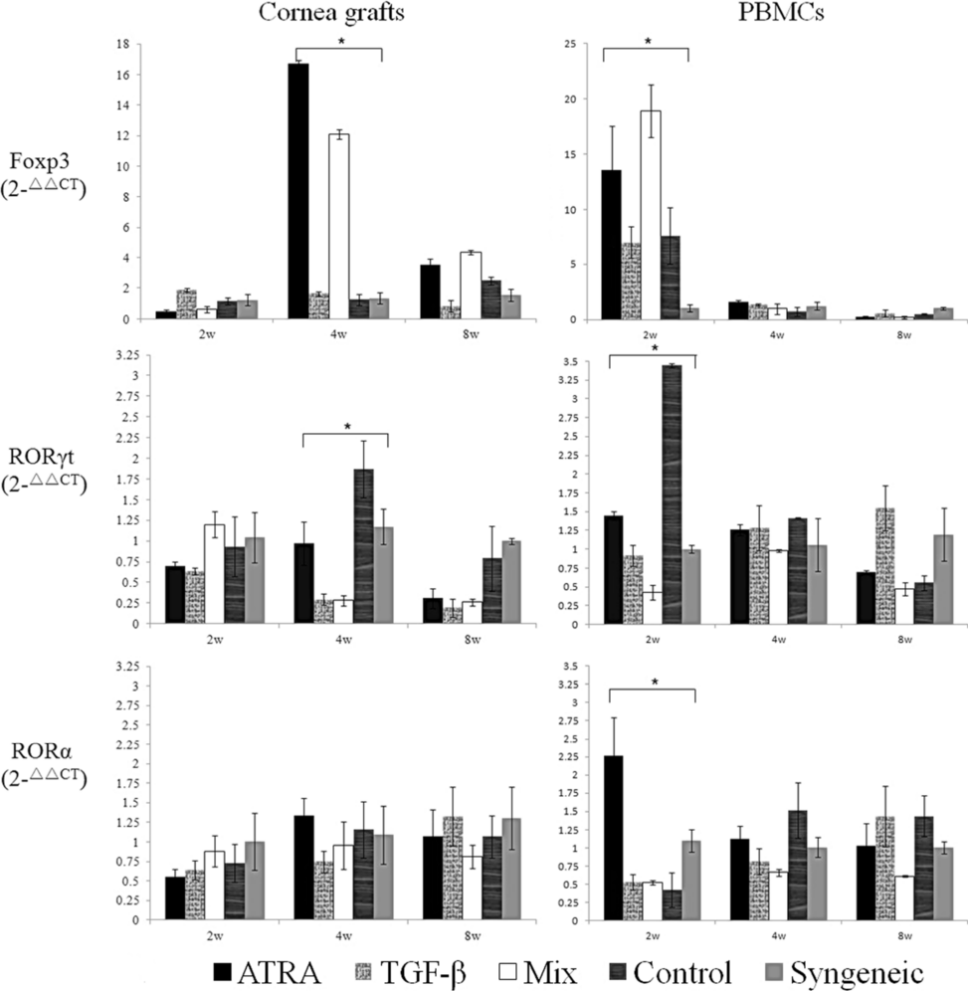
**Gene expression of Foxp3, RORγt and RORα in the grafts and PBMCs.** At 2wks postsurgery, a mixed treatment of ATRA and TGF-β significantly promoted the Foxp3 expression in PBMCs and suppressed the RORγt expression as well. At 4wks, the Foxp3 expression increased significantly in the allogeneic grafts either with a mixed treatment or ATRA alone, and the reduced expression of RORγt was likewise found in these two groups. At each time point, each group contained at least five corneas and three blood samples. Data are presented as the mean value ± standard error and are representative of at least two independent experiments. * P < 0.05.

At 2wks postoperative, the Foxp3 gene expression in PBMCs was significantly higher in recipients treated with either ATRA or the combination of ATRA and TGF-β (P = 0.023), while at 4wks postoperative, the Foxp3 expression in the cornea grafts increased significantly in these two groups (P = 0.012). A significantly lower RORγt expression in the PBMCs was found in those receiving a mixed treatment at 2wks postoperative (P = 0.042), while the RORγt expression in the grafts was significantly lower in recipients treated with either TGF-β or the combination of ATRA and TGF-β at 4wks (P = 0.025). The RORα expression in PBMCs was significantly higher in recipients treated with ATRA at 2wks (P = 0.029). As for RORα expression in grafts, no statistical significance was found among the five groups, as shown in Figure 6.

### A mixed treatment of ATRA and TGF-β reduces the IL-17 level in the serum and aqueous humor and increases the IL-10 level in the aqueous humor

**Figure 7:**
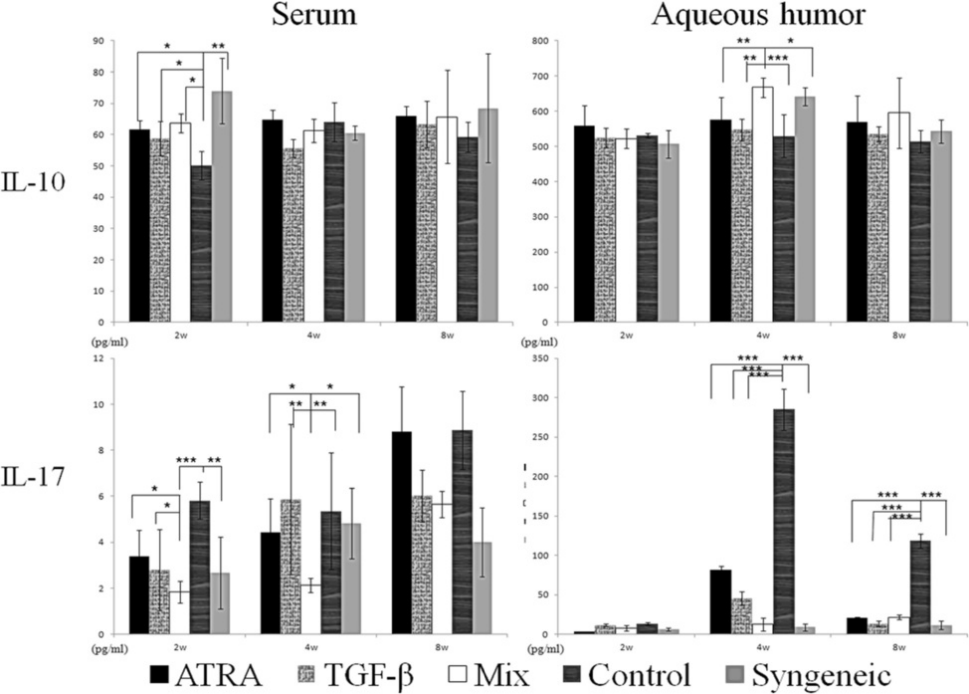
**The concentration of IL-10 and IL-17 in the serum and aqueous humor.** A mixed treatment of ATRA and TGF-β reduced the serous level of IL-17 in serum at 2wks and 4wks and decreased the concentration of IL-17 in the aqueous humor at 4wks and 8wks. Meanwhile, a mixed treatment increased the IL-10 level in the aqueous humor at 4wks. At each time point, each group contained at least three samples of serum and five samples of aqueous humor. Data are presented as the mean value ± standard error and are representative of at least two independent experiments. * P < 0.05, ** P < 0.01, ***P < 0.001.

Figure 7 showed that at 2wks postoperative, the serous level of IL-10 was (50.1 ± 4.6) pg/ml in the recipients treated with a control solution, which is significantly lower than the other four groups (F = 8.063, P = 0.011). At 4wks, the combined treatment of ATRA and TGF-β significantly increased the level of IL-10 in the aqueous humor as compared with the other groups (F = 7.505, P = 0.002). At 2wks and 4wks, the serous level of IL-17 was (1.8 ± 0.7) pg/ml and (2.1 ± 1.3) pg/ml in recipients with a mixed treatment, which is significantly lower than recipients with any other treatments (F = 5.887 and 6.453, P = 0.005 and 0.004). At 4wks and 8wks, the concentration of IL-17 in the aqueous humor was far higher in the recipients treated with a control solution than the other groups (F = 682.622 and 248.868, both P < 0.001).

### A mixed treatment of ATRA and TGF-β increases the suppressive activity of CD4 + CD25 + Foxp3+ Tregs *in vitro*

**Figure 8:**
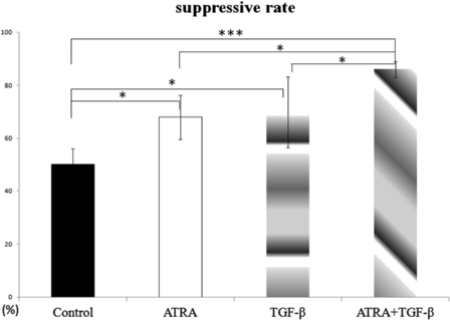
**The suppressive activity of CD4 + CD25 + Foxp3+ T cells isolated from recipient mice.** A single treatment of either ATRA or TGF-β could enhance the suppressive activity of CD4 + CD25 + Foxp3+ T cells *in vitro.* A mixed treatment of ATRA and TGF-β could further increase the suppressive activity. Each group contained five samples. Data are presented as the mean value ± standard error and are representative of at least three independent experiments. * P < 0.05, ***P < 0.001.

Figure 8 illustrated that the suppressive rate of Tregs isolated from recipient mice treated with a control solution was (50.1 ± 6.1)%. Administration of either ATRA or TGF-β increased the suppressive rate to (67.9 ± 8.2)% and (69.9 ± 13.4)%, respectively. A mixed treatment of both agents could further increase the suppressive rate to (86.1 ± 3.0)%, which was significantly higher than the control group (F = 10.842, P = 0.002).

## Discussion

Th17 cells, which recruit monocytes and neutrophils by the secretion of IL-17 and suppress the expansion of regulatory T cells, have been implicated to be predominantly involved in the acute phase of allograft rejection [8,17,18], especially within 5–14 days. This study confirmed that Th17 cells were detrimental to allogeneic graft survival. Moreover, we not only found the enhancement of Th17 cells in peripheral blood and draining lymph nodes at the early stage of the rejection process after corneal transplantation, but also identified an increased number of IL-17-positive cells in conjunctiva, enhanced expression of RORγt in cornea grafts and an elevated level of IL-17 in the aqueous humor at the late stage of rejection process, which has not been reported by previous studies. We assumed that the disparities might be attributed to two reasons. First, unlike other solid organ transplantation, immunological rejection after allogeneic corneal transplantation does not affect the survival of the recipients, which is conducive to observing the long-term dynamic changes of Th17 cells. Second, the activated Th17 cells might take some time to migrate from peripheral blood and lymphoid tissue into conjunctiva and further into cornea graft, since cornea is absent of blood and lymphatic vessels.

Although CD4 + CD25 + Foxp3 + Tregs have strong immunosuppressive potency, they are present in very low numbers in a normal host and have an anergic phenotype. The upregulation of CD4 + CD25 + Foxp3 + Tregs induced by certain agents, such as rapamycin and IL-2, has been proven to protect the allogeneic graft and prolong graft survival [19]. Nevertheless, the expression of Foxp3 is unstable and will be down-regulated in an IL-6-rich, Th17-promoting environment. When such Treg ‘deprogramming’ occurs, Tregs fail to exert their original immunoinhibitory role [20,21] and may convert into competent Foxp3- effector T cells, which up-regulate the RORγt expression [11]. Inflammation-dampening strategies, particularly manipulating the inflammation occurring in the graft and draining lymph nodes, may guide the host immunity from an adverse effect toward tissue-protective phenotypes. Therefore, shifting the Th17-Treg profile toward Tregs and maintaining the Foxp3 expression is crucial in protecting the allografts and achieving graft acceptance.

It has been shown that ATRA is one of potential therapeutic agents capable of skewing the Treg–Th17 axis away from a Th17 response. Retinoic acid was first identified as a potential regulator of T cell homing to the intestinal tract, with intestinal dendritic cells being unique in their production of retinoic acid [22]. A novel role of retinoic acid in the regulation of the Th17–Treg axis, inhibiting the development of Th17 cells by splenic dendritic cells, was discovered by Mucida et al. [23] Recent studies have demonstrated that the reduction in IL-17 was accompanied by a reduction in the RORγt expression in response to ATRA [24]. Furthermore, Tregs expanded with ATRA are resistant to the inhibitory effects of IL-6 on the Foxp3 expression and prevent Th17 conversion [15], thus stabilizing the Tregs population and function.

However, ATRA is not sufficient to convert mouse naïve T cells undergoing activation into Foxp3+ T cells. Many studies have confirmed that the TGF-β signal is additionally required for a reliable expression of Foxp3 in T cells *in vitro* [12,23,24]. Some findings suggested that ATRA enhanced TGF-β signaling by increasing the expression and phosphorylation of Smad3 and also inhibited the expression of IL-6 receptor α chain [24,25]. Other findings suggested that ATRA reduced the STAT6 binding to the Foxp3 promoter or indirectly relieved inhibition of the TGF-β-mediated Foxp3 expression from cytokine-producing CD4^+^CD44^hi^ T cells and finally enhanced the Foxp3 expression [26,27]. A few *in vivo* studies also confirmed that with the presence of TGF-β, ATRA promoted Treg conversion and inhibited Th17 induction [18,28].

The combined administration of ATRA and TGF-β has been proven to attenuate heart, liver and trachea rejection [18,29,30]. The effect was attributed, at least partially, to the shifted balance of the Th17-Treg axis, meaning for the conversion of the CD4 + Foxp3 + Tregs and suppressed induction of the Th17 cells. This study found that with the presence of TGF-β, ATRA could maximally promote allogeneic graft survival. In contrast, the treatment of ATRA alone could not entirely mitigate graft rejection. Previous studies have demonstrated that without TGF-β, ATRA could not regulate Tregs conversion but retained the ability to inhibit IL-17 production [15,16], which might be the possible explanation for the finding.

The overexpression of RORγt and RORα is characterized in the differentiation of Th17 cells [31,32]. RORγt has been proposed as a ‘master regulator’ for Th17 differentiation, while RORα seems to synergize with RORγt to promote Th17 differentiation. It has been demonstrated that coexpression of RORγt and RORα synergistically enhances the number of IL-17-producing cells [31]. However, this study found that the combined administration of ATRA and TGF-β inhibits the expression of RORγt rather than RORα, especially in the allograft. Taking into consideration that RORα is not the major player in the induction of Th17 cells and that ATRA might regulate Th17 differentiation through other mechanisms [23,33], the exact effect of ATRA on RORα merits further investigation.

Our results showed an elevated level of IL-17 in the aqueous humor at 4wks and 8wks postoperative. Accordingly, a slit lamp and a histological examination at the same time points revealed graft angiogenesis and fibrous tissue proliferation. IL-17, the key cytokine produced by Th17 cells, has been proven to recruit monocytes and neutrophils, promote angiogenesis [34], and stimulate the proliferation of fibroblasts [35]. Many studies have confirmed that IL-17 is involved in allograft rejection, and neutralization of IL-17 inhibits accelerated allograft rejection. In contrast, IL-10, one of the most important immunosuppressive cytokines secreted by Tregs, has been proven to favor allograft acceptance and has a close relationship with the allograft’s function [36,37]. Various studies have reported that after solid organ transplantation, an elevated content of IL-10 was found among patients with grafts accepted or without acute rejection episodes [36,37], confirming its immunomodulatory effects. The combined treatment of ATRA and TGF-β could promote IL-10 levels in the serum and aqueous humor and reduce IL-17 expression simultaneously, and changes of these cytokines are consistent with the prognosis of cornea grafts. Combining our results and previous findings, we deduce that although reducing the Th17 generation and manipulating host inflammation may well be beneficial for the outcome of allograft, fostering regulatory-type alloimmunity is also essential to achieve lasting transplant tolerance and improve function posttransplant. Nevertheless, the statistical differences for the level of IL-10 are very small in this research and may not be biologically important. The exact role of IL-10 in modulating immunological reaction toward allogeneic corneal grafts merits further studies.

Apart from the effect on the Treg-Th17 axis, another beneficial outcome of ATRA to graft survival should also be taken into consideration. It has been shown that the recovery of corneal epithelial cells is important to graft survival after corneal transplantation. Delayed epithelium recovery or persistent epithelial defect would hamper or even be detrimental to graft survival [38]. ATRA has been shown to promote corneal epithelium wound healing [39]. This study found that either single administration of ATRA or combined treatment of ATRA + TGF-β could improve graft survival at early time point after transplantation, indicating that the beneficial effect of ATRA to corneal epithelium recovery might also play an important role in promoting graft survival.

## Conclusions

In brief, with the presence of TGF-β, the administration of ATRA not only promoted graft survival in recipient mice after allogeneic penetrating keratoplasty, but also simultaneously expanded the CD4 + CD25 + Foxp3+ Tregs and reduced the population of Th17 cells in the peripheral blood, spleen and draining lymph nodes. Our findings suggest that the combined application of ATRA and TGF-β may shift the Th17-Treg balance toward Tregs, hence facilitating the induction of immunological tolerance after allogenic corneal transplantation and representing a potential therapeutic approach in the treatment of posttransplant rejection.

## Methods

### Mice

Male BALB/c (H-2d) and C57BL/6 (H-2b) mice (weighing 20-24 g, 6-8-weeks-old) were purchased from the Department of Laboratory Animal Science, Fudan University. All mice were housed in a specific pathogen-free (SPF) environment. All experimental manipulations were undertaken in accordance with the Institutional Guidelines for the Care and Use of Laboratory Animals (Additional file 1) and ARVO Statement for the Use of Animals in Ophthalmic and Vision Research.

### Orthotopic corneal transplantation (penetrating keratoplasty)

The surgical procedure was performed as previously reported [19]. In brief, C57BL/6 mice were sacrificed by cervical dislocation and a 2-mm-diameter trephine was used to obtain the cornea as the donor of allogenic penetrating keratoplasty. BALB/c mice, with the right eyes serving as the recipients, were anesthetized with 100 mg/Kg of ketamine hydrochloride and 5 mg/Kg of diazepam. After dilating the pupil by administering 0.25% tropicamide eye drops, a 1.5-mm-diameter trephine was used to cut the recipient cornea at the depth of 80-90%, and then paracentesis was carefully done with a 1 ml needle to avoid damaging the lens. Viscoelastic material containing 3% sodium hyaluronate (Healon, Advanced Medical Optics, Santa Ana, CA) was injected immediately to maintain the anterior chamber. Then, the recipient cornea was cut with scissors and the donor cornea was fixed to the recipient’s bed with eight interrupted 11–0 nylon sutures. Erythromycin eye ointment was administered in the conjunctival sac after the surgery, and then the eyelids were sutured with an 8–0 mattress sutures. The eyelids sutures were removed at day 3 post-operatively and the corneal sutures were removed 7 days after surgery. Simultaneously, the syngeneic penetrating keratoplasty was performed from BALB/c to BALB/c mice, using the completely same surgical procedure.

### Post-transplant therapies

A total of 120 mice underwent allogeneic penetrating keratoplasty, and the recipient mice were randomly divided into four groups, each containing 30 mice. Group A was intraperitoneally administered with ATRA (Sigma-Aldrich) at a dose of 3 mg/kg/day, which was dissolved in dimethyl sulfoxide (DMSO). Group B was intraperitoneally treated with TGF-β 5 μg/kg/day (PreproTech, NJ, USA). The combined treatment of ATRA 3 mg/kg/day and TGF-β 5 μg/kg/day was administered to group C. The recipient mice in group D received an equivalent volume of vehicle as control. Group E contained another 30 syngeneic graft recipient mice, which didn’t receive any treatment. If mice were dead during the follow-up, we would recruit the same number of recipient mice to assure that at least 10 mice in each group could be sacrificed for further *in vitro* experiments at 2wks, 4wks and 8wks after surgery.

### Assessment of the grafts

All recipient mice were examined under the slitlamp biomicroscope two times a week after surgery, and digital photographs of the cornea were taken using a Canon 8-megapixel digital camera attached to the slitlamp biomicroscope. The transparency of the graft and the formation of neovascularization in the graft and host tissue were recorded and scored to assess the degree of graft rejection according to the previous literature [40]. Mice with infection, hyphema and cataract were excluded.

### Flow cytometric analysis

FITC-, APC- or PE-conjugated antibodies specific for mouse CD4 (RM4-5), CD25 (PC61.5), Foxp3 (FJK-16 s), CD3e (145-2C11), CD8a (53–6.7), IL-17 (TC11-18H10.1), and their isotype controls were purchased from eBioscience, or BioLegend (San Diego, CA, USA).

At 2wks, 4wks and 8wks after surgery, four recipient mice in each group were sacrificed and the peripheral blood was collected in heperanized tubes. Meanwhile, the spleen and draining lymph nodes were obtained. Single cell suspension of the spleen and lymph nodes was prepared by compression with the plunger of a 3 ml syringe. After that, the cells were centrifuged and resuspended in an appropriate volume of flow cytometry staining buffer so as to adjust the final cell concentration to 2x10^7^/mL. Each sample was then divided into two parts. One was incubated with anti-mouse CD4 and CD25 mAb for at least 30 min at 4°C in the dark, and then washed, fixed, permeabilized and stained with anti-mouse Foxp3 mAb for another 30 min at 4°C in the dark. The other part was stimulated with 0.1 μg/mL PMA (Sigma), 1 μg/mL ionomycin (Sigma) and monensin (GolgiStop, 1 μl/ml; BD Biosciences) for 4 hrs at 37°C. Following that, it was incubated with anti-mouse CD3 and CD8 mAb for at least 30 min at 4°C in the dark and then washed, fixed, permeabilized and stained with anti-mouse IL-17 mAb for another 30 min at 4°C in the dark. Finally, the cells were resuspended with a flow cytometry staining buffer, and analyzed using an FACS Calibar flow cytometer (Becton Dickinson, San Jose, CA) and CellQuest software.

### Histology assessment

At 2wks, 4wks and 8wks after surgery, three recipient mice in each group were sacrificed. The eyeballs were enucleated and fixed in the mixture of 40% formaldehyde, 40% glacial acetic acid and 95% ethanol for 24 hrs and then dehydrated in graded ethanol solutions and embedded in paraffin wax. The embedded tissue was cut to 4–6 micron-thick sections. The sections for histological examination underwent routine hematoxylin eosin staining and were studied under light microscopy (Nikon, ALPHAPHOTO-2 YS2, Japan). The quantitative analysis of infiltrated inflammatory cells was performed by a masked observer using software ImageJ (http://rsb.info.nih.gov/ij/download.html).

### Immunofluorescence staining

To perform immunofluorescence staining, the sections were firstly deparaffinized in xylenes using three changes for 5 min each and then hydrated gradually through graded alcohols: wash in 100% ethanol twice for 10 min each and then 95% ethanol twice for 10 min each. Antigen retrieval was performed by heating the slides in 10 mM sodium citrate buffer, pH 6.0, at 95°C for 5 min and then cooling them in the buffer for approximately 20 min until they reached room temperature. Afterward, the slices were washed with PBS three times and the excess liquid was aspirated. After several blocking steps, the sections were incubated with 1:100 rabbit anti-mouse Foxp3 mAb (H-190, Santa Cruz Biotechnology, Inc., Santa Cruz, CA) and 1:100 rat anti-mouse IL-17 mAb (TC11-18H10, Santa Cruz) at 37°C for 1 hr and then at 4°C overnight. The slices were washed with PBS three times for 5 min each and then incubated with Alexa Fluor 546 donkey anti-rabbit IgG (Invitrogen, CA, USA) and Alexa Fluor 488 rabbit anti-rat IgG (Invitrogen) for 2 hrs. Finally, the sections were mounted with glycerol and examined under laser scanning confocal microscopy (TCS SP2, Leica, Germany). The quantitative analysis of Foxp3+ cells and IL-17+ cells was also performed as mentioned above.

### Real-time PCR of Foxp3, RORγt and RORα in the grafts and PBMCs

At 2wks, 4wks and 8wks after surgery, three recipient mice in each group were sacrificed. PBMCs were isolated on Ficoll-Hypaque density gradients (GE Healthcare Bio-Sciences Corp., Piscataway, NJ). Cells recovered from the gradient interface were washed twice in PBS and then collected. Meanwhile, the eyeballs were enucleated and the cornea was harvested from the limbus. The gene expression of Foxp3, RORγt and RORα in the cornea tissue and PBMCs was analyzed using quantitative real-time PCR. Total RNA was extracted from homogenized harvested cornea grafts using the Qiagen RNeasy mini Kit (Qiagen, Tokyo, Japan), according to the manufacturer’s instructions. Reverse transcription to cDNA was performed using Taqman reverse transcription reagents (Applied Biosystems, USA). The sequences of forward and reverse primer for Foxp3 gene were 5′ CTATGCCACCCTTATCCGA 3′ and 5′ TCCTCTTCTTGCGAAACTCA 3′, respectively. The sequences for RORγt gene were 5′ CAGGAGCAATGGAAGTCGTC 3′ and 5′ CCGTGTAGAGGGCAATCTCA 3′. Those for RORα were 5′ GCTGTGTGCCATCAAGATTAC 3′ and 5′ CACGGTGTTGTTCTGAGAGTC 3′. Mouse β-actin was used as an endogenous control. Relative quantification assays for gene expression of Foxp3, RORγt and RORα were performed using a StepOnePlus real-time PCR system (Applied Biosystem, USA). For each example, the threshold cycle (CT) value of the target gene was normalized using the formula △CT = CT_target gene_-CT_β-actin_. △△CT of each target gene was further calculated by the formula △CT_treated group_-△CT_syngeneic group_. The mean △△CT was determined and the relative mRNA expression of Foxp3, RORγt and RORα was calculated with 2^-△△CT^.

### ELISA

The peripheral blood was collected under ethyl ether inhalation anesthesia, and the aqueous humor was obtained with capillary glass tube right after the sacrifice of recipient mice. An enzyme-linked immunosorbent assay was performed to test the expression of IL-10 and IL-17 in the serum and aqueous humor, using ELISA kits (M1000 and M1700, R&D System, MN, USA).

### Immunosuppressive assay

The immunosuppressive activity of CD4 + CD25 + Foxp3+ Tregs was determined by the suppression of a mixed lymphocyte reaction (MLR). Splenocytes (1 × 10^5^/well) from naive BALB/c mice were either cultured alone as a negative control group or cocultured with mitomycin (25 mg/L)-pretreated splenocytes (1 × 10^5^/well) from C57BL/6 as a positive control group. The CD4 + CD25 + Foxp3+ Tregs were isolated by MACS with a CD4 + CD25+ Regulatory T Cell Isolation Kit (Miltenyi Biotec, Germany) from recipient mice treated with ATRA, TGF-β, ATRA+ TGF-β or a control solution. Isolated Tregs (0.5 × 10^5^/well) were either added to the coculture system and cultured for 3 days as the experimental group or cultured alone as a blank control group. A non-radioactive cell proliferation assay (G4000, Promega, WI, USA) was used to determine the cell proliferation activity, according to the manufacturer’s instructions. The absorbance at 570 nm (A value) was recorded by a microtiter plate reader. The suppressive rate was calculated as: suppressive rate = [1 − (A_experimental group_ − A_blank control group_ − A_negative control group_)/ (A_positive control group_ − A_negative control group_)] × 100 %.

### Statistical analysis

Data are presented as the mean ± SD. Statistical significance was determined using One-way analysis of variance (ANOVA), Student’s t-test or Kruskal-Wallis test. For each ANOVA, post-hoc Fisher PLSD (protected least significant difference) was performed to determine each individual group difference. A Kaplan-Meier survival curve and Log-rank statistical analysis were used to compare the graft survival rate among the five groups. All tests were considered statistically significant at P <0.05 (SPSS for Windows, version 13.0; SPSS, Inc., Chicago, IL).

## Additional file

Additional file 1:
**Animal ethics statement.**

## Abbreviation

ATRA: All-*trans* retinoid acid
TGF-β: Transforming growth factor beta
Treg: Regulatory T cells
IL: Interleukin
Th17: Interleukin 17 -producing T helper cells
PBMCs: Peripheral blood mononuclear cells
