# Supplementary figures and images for: All-trans retinoid acid promotes allogeneic corneal graft survival in mice by regulating Treg-Th17 balance in the presence of TGF-β
Source: BMC Immunol. 2015 Mar 19;16:17. doi: 10.1186/s12865-015-0082-3 (PMC4395899; doi:10.1186/s12865-015-0082-3)

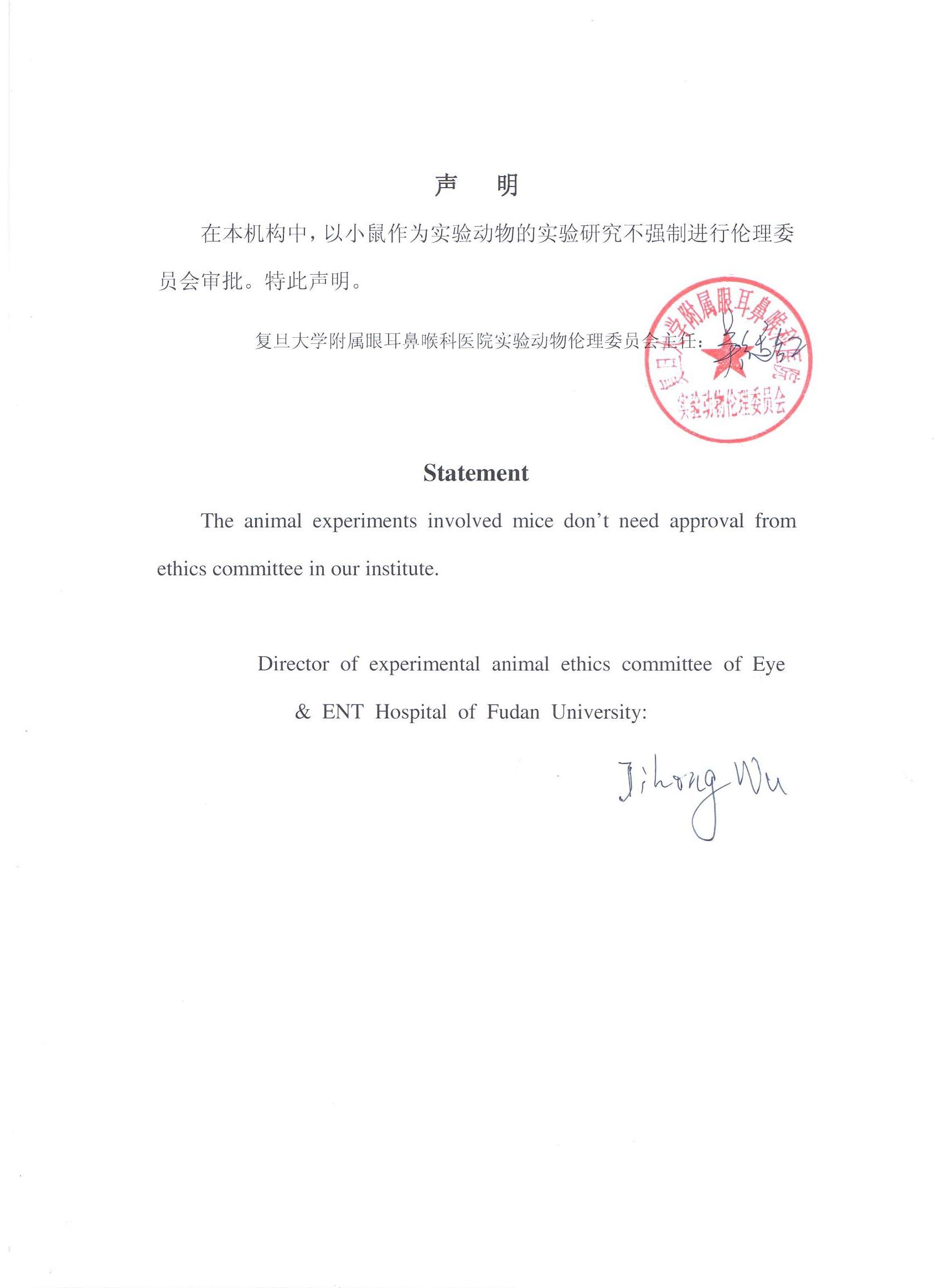

Supplement: Additional file 1: — Animal ethics statement. [file 12865_2015_82_MOESM1_ESM.jpeg]
